# The Predictive Value of Serum Calcium on Heart Rate Variability and Cardiac Function in Type 2 Diabetes Patients

**DOI:** 10.3389/fendo.2022.864008

**Published:** 2022-04-14

**Authors:** Junyi Wang, Zihui Xu, Kang Lv, Yingchun Ye, Deng Luo, Li Wan, Fen Zhou, Ailin Yu, Shuo Wang, Jingcheng Liu, Ling Gao

**Affiliations:** ^1^ Department of Endocrinology & Metabolism, Renmin Hospital of Wuhan University, Wuhan, China; ^2^ Shenzhen University, College of Big Data and Internet, Shenzhen, China

**Keywords:** calcium, heart rate variability, type 2 diabetes mellitus, SDNN, cardiovascular autonomic neuropathy

## Abstract

**Background:**

Cardiovascular autonomic neuropathy (CAN) is common in patients with type 2 diabetes mellitus (T2DM), mainly presented as decreased heart rate variability (HRV) which often leads to cardiac death. However, HRV measurement is not convenient in most clinics. Therefore, identifying high-risk patients for CAN in diabetes with easier measurements is crucial for the early intervention and prevention of catastrophic consequences.

**Methods:**

In this cross-sectional study, 675 T2DM patients with normocalcemia were selected. Of these, they were divided into two groups: normal HRV group (n = 425, 100 ms≤ SDNN ≤180 ms) *vs*. declined HRV group (n = 250, SDNN <100 ms). All patients’ clinical data were collected and the correlation of clinical variables with HRV were analyzed by correlation and logistic regression analysis. The area below the ROC curve was used to evaluate the predictive performance of serum calcium on HRV.

**Results:**

In this study, declines in HRV were present in 37.0% of T2DM patients. Significant differences in albumin-adjusted serum calcium levels (CaA) (8.86 ± 0.27 *vs.* 9.13 ± 0.39 mg/dl, *p <*0.001) and E/A (0.78 ± 0.22 *vs.* 0.83 ± 0.26, *p* = 0.029) were observed between declined HRV and normal HRV groups. Bivariate linear correlation analysis showed that CaA and E/A were positively correlated with HRV parameters including SDNN (*p* < 0.001), SDNN index (*p* < 0.001), and Triangle index (*p* < 0.05). The AUC in the ROC curve for the prediction of CaA on HRV was 0.730 (95% CI (0.750–0.815), *p* < 0.001). The cutoff value of CaA was 8.87 mg/dl (sensitivity 0.644, specificity 0.814). The T2DM patients with CaA <8.87 mg/dl had significantly lower HRV parameters (SDNN, SDNN index, rMSSD, and triangle index) than those with CaA ≥8.87 mg/dl (*p* < 0.01, respectively). Multivariate logistic regression analysis showed a significantly increased risk of declined HRV in subjects with CaA level <8.87 mg/dl [OR (95% CI), 0.049 (0.024–0.099), *p* < 0.001].

**Conclusions:**

Declined HRV is associated with a lower CaA level and worse cardiac function. The serum calcium level can be used for risk evaluation of declined HRV in T2DM patients even within the normocalcemic range.

## Background

Type 2 diabetes mellitus (T2DM) has become one of the major chronic diseases in the world. Besides macrovascular and microvascular complications, T2DM is also commonly complicated with cardiovascular autonomic neuropathy (CAN) (1, 2). CAN, characterized by increased sympathetic activity and decreased parasympathetic activity, can be evaluated by heart rate variability (HRV) obtained from ambulatory electrocardiogram (3–5). Moreover, reduced HRV is considered to be a risk marker of cardiovascular death and also a leading cause of T2DM death (6, 7). Although HRV has been suggested to be highly associated with the onset and development of CAN in diabetes (8–10), more convenient measures to identify high-risk CAN patients in T2DM for early prevention are needed.

It is well known that serum calcium affects cardiac electrical activity and cardiac contraction. Evidence demonstrated that hypocalcemia led to an increase in ventricular action potential duration and prolongation of the QTc interval, which is associated with increased risk of arrhythmias (11–13). On the other hand, patients with hypercalcemia had a shortened QTc interval (12, 13). Moreover, a decrease in serum calcium level was associated with increased risk of sudden cardiac arrest (14) and an increase in serum calcium level was associated with an increased risk of T2DM (15–18). However, the relationship between serum calcium and CAN in diabetes is not certain. The aim of this study was to assess the predictive value of serum calcium (within normal range) on CAN in T2DM patients.

## Methods

### Participants

675 T2DM patients who visited the Renmin Hospital of Wuhan University for evaluation or treatment from January 2019 to June 2021 with normocalcemia (serum calcium in the reference range of 8.46–10.10 mg/dl) were recruited. The exclusion criteria for patients were (1) dilated cardiomyopathy or hypertrophic cardiomyopathy; (2) pacemaker implantation; (3) degree II and above atrioventricular block, atrial fibrillation, sick sinus syndrome, and frequent premature contraction; (4) glomerular filtration rate <60 ml/min or urine albumin per gram urine creatinine (Alb/Cr) >300 mg/g; (5) alanine aminotransferase >120 U/l; (6) parathyroid disease or vitamin D-related disorders; (7) medication history including bisphosphonate, vitamin D, and diuretics which may influence calcium within the past 1 month; (8) serum calcium out of reference range (8.46–10.10 mg/dl); and (9) malignant tumor.

The patients’ smoking history, drinking history, and current and past medical history were collected. T2DM was diagnosed according to the World Health Organization criteria (19). This study was approved by the ethical review board of Renmin Hospital of Wuhan University and complied with the Helsinki declaration.

### Biochemical Measurements

A 12-h overnight fasting venous blood sample was collected in all subjects. The serum electrolytes (calcium, sodium, potassium, chlorine, phosphate, and magnesium), uric acid, creatinine, albumin (ALB), total cholesterol (TC), triglycerides (TG), low-density lipoprotein cholesterol (LDL-C), high-density lipoprotein cholesterol (HDL-C), high-sensitivity C-reactive protein (hs-CRP), NT-proBNP, and fasting plasma glucose (FPG) were measured by a biochemical autoanalyzer (Abbott C8000, Chicago, IL, USA). HbA1c and homocysteine (HCY) were measured by high-performance liquid chromatography (HPLC; Bio-Rad, Hercules, CA, USA).

Serum calcium CV_w_ (within-subject coefficient of variation) = 2.1 mmol/l; CV_b_ (between-subject coefficient of variation) =2.5 mmol/l; imprecision = 1.1%; bias = 0.8%; TEa (total allowable error, *p* < 0.05) = 2.5%. Albumin-adjusted serum calcium levels (CaA) (mg/dl) were derived from formula (serum total calcium concentration (mg/dl) + 0.8 × [4 − serum albumin concentration (g/dl)] (17) to adjust total calcium for hypoalbuminemia to permit approximation of the ionized calcium, which is physiologically active and under homeostatic control. The fasting triglyceride-glucose index (TyG), which was sensitive for recognizing insulin resistance, was calculated using the formula: Ln [TG (mg/dl) × FPG (mg/dl)/2] (20).

### Ambulatory Electrocardiogram Monitoring

24-hour ambulatory electrocardiogram (ECG) was recorded using a wearable 12-lead digital Holter device (JincoMed, Beijing, China) with a data acquisition speed of 4,000 Hz. Subjects were told to follow their daily routines but to avoid intense physical activities or shower. ECG data were processed using a professional Holter analysis system developed by JincoMed, including algorithms for QRS labeling, arrhythmia detection, artifact identification, and data correction, followed by manual review by Holter technicians in Renmin Hospital of Wuhan University. Both time- and frequency-domain HRV parameters were derived from ambulatory ECG data. Time-domain HRV parameters quantify the variability of successive heartbeat interval, including standard deviation of normal R–R intervals (SDNN), mean standard deviation of normal R–R intervals for 5-min segments within 24 h (SDNN index), root mean square of successive RR interval differences (rMSSD), and the integral of the density of the RR interval differences (triangular index). Frequency-domain parameters estimate the distribution of power into different frequency bands, including high-frequency (HF, 0.15–0.40 Hz), low-frequency (LF, 0.04–0.15 Hz), and LF/HF ratio. 24-hour and hourly averages of time-domain HRV measures and hourly averages of frequency domain measures were obtained.

### Grouping

SDNN is widely used to evaluate autonomic function and is considered to be a sensitive indicator in T2DM patients (21). In this study, T2DM subjects were divided into two groups, declined HRV (SDNN <100 ms) and normal HRV (100 ms ≤ SDNN ≤180 ms), according to heart rate variability standards recommended by the European Society of Cardiology and the North American Society of Pacing and Electrophysiology Task Force (22).

### Echocardiography

Echocardiographic examinations were performed using commercially available ultrasound diagnostic instruments (GE Vingmed Ultrasound, Horten, Norway) in accordance with the guidelines issued by the American Society of Echocardiography (23). We measured cardiac structure indicators, which included left atrial diameter (LAD), aortic root dimension (AOD), left ventricular end diastolic dimension (LVDd), diastolic interventricular septum thickness (IVSd), main pulmonary artery diameter (MPAD), right ventricular end diastolic diameter (RVDd), right atrium diastolic transverse diameter (RADd), and diastolic left ventricular posterior wall thickness (LVPWd).

The Doppler spectrum of the mitral valve pulse was recorded in an apical four-chamber view. The peak velocity of the filling peak in the early diastolic period (E) and late diastolic period (A) and the early diastolic mitral annulus velocity (e′) were measured. Cardiac diastolic function was assessed by E/A ratio and E/e′ ratio. Three cardiac cycles were measured, and the average value was used. Diastolic dysfunction was defined as E/A ratio <1.0 or E/e′ ratio >15 (24). Left ventricular systolic function was assessed by left ventricular ejection fraction (LVEF).

### Statistical Analysis

Continuous variables were presented as mean ± standard deviation (SD), as well as frequencies and percentages for categorical variables. Continuous variables with non-normal distribution are represented by median (M) and quartile range (QR). Differences in normally distributed variables were determined by independent-sample T test or one-way ANOVA. Chi-square tests were applied for categorical variables. Bivariate linear correlation (Pearson correlation) analysis was carried out to evaluate the associations between CaA and HRV parameters.

Logistic regression analysis was performed using HRV as the dependent variable to analyze the association between CaA and HRV after adjusting for potential confounders. Odds ratios (OR) with 95% confidence intervals (CI) were calculated for the relative risk of lower serum calcium level with declined HRV. The ability to predict declined HRV of CaA was evaluated using the area under the curve (AUC) in the receiver operating characteristic (ROC) curve. All statistical analysis were performed using SPSS version 22.0. All tests were two-sided, and *p <*0.05 was considered statistically significant.

## Results

### Clinical Characteristics

In this study, 675 T2DM subjects (399 men and 276 women) were included, with a mean age of 62.49 ± 11.36 years (from 23 to 95 years old). Declined HRV (SDNN <100 ms), hypertension, coronary heart disease, and carotid atherosclerosis were present in 250 (37.0%), 501 (74.2%), 370 (54.8%), and 132 (71.4%) patients, respectively. There were no individuals of SDNN >180 ms in the enrolled T2DM patients.

Significant differences in CaA (8.86 ± 0.27 *vs*. 9.13 ± 0.39 mg/dl, *p <*0.001) were observed between declined HRV and normal HRV groups (**Table 1**). The patients with declined HRV were older and had higher levels of HbA1c, NT-proBNP, and hs-CRP, as well as lower levels of HDL-C and LDL-C than those with normal HRV (*p* < 0.05, respectively). There was no significant difference in prevalence of smoking or drinking history, hypertension, coronary heart disease, and carotid atherosclerosis between the two groups (**Table 1**).

**Table 1:** Baseline characteristics of subjects categorized by SDNN.

Characteristics	HRV		*p*
	SDNN <100 ms	100 ms≤ SDNN ≤180 ms	
	(n = 250)	(n = 425)	
Age (years)	64.36 ± 11.05	61.39 ± 11.41	0.001
Male, n (%)	140 (56.0)	259 (60.9)	0.207
Smoking, n (%)	73 (29.2)	97 (22.8)	0.065
Drinking, n (%)	60 (24.0)	84 (19.8)	0.195
Hypertension, n (%)	178 (71.2)	323 (76.0)	0.148
Coronary heart disease, n (%)	145 (58.0)	225 (52.9)	0.182
Carotid atherosclerosis, n (%)	56 (75.7)	76 (68.5)	0.288
FPG [M(QR), mmol/L]	8.09 (3.59)	7.52 (3.01)	0.073
TyG index	9.17 ± 0.74	9.07 ± 0.71	0.079
HbA1c (%)	7.99 ± 1.42	7.58 ± 1.50	0.001
Uric acid (μmol/L)	343 ± 99	349 ± 96	0.483
Creatinine (μmol/L)	65.9 ± 16.7	64.8 ± 15.5	0.400
ALB (g/L)	40.89 ± 3.69	41.05 ± 3.68	0.581
TG (mmol/L)	1.87 ± 1.22	1.80 ± 1.17	0.460
TC (mmol/L)	4.03 ± 1.04	4.20 ± 1.14	0.065
HDL-C (mmol/L)	0.96 ± 0.26	1.03 ± 0.27	0.002
LDL-C (mmol/L)	2.31 ± 0.92	2.40 ± 0.88	0.030
K [M(QR),mg/dl]	15.56 (2.02)	15.48 (1.70)	0.560
Na (mg/dl)	324.83 ± 8.11	325.59 ± 7.66	0.223
Cl (mg/dl)	375.26 ± 10.78	376.44 ± 10.78	0.192
P (mg/dl)	3.58 ± 0.58	3.56 ± 0.60	0.708
Mg (mg/dl)	1.93 ± 0.21	1.95 ± 0.20	0.229
Ca (mg/dl)	9.00 ± 0.64	9.30 ± 0.67	<0.001
CaA (mg/dl)	8.86 ± 0.27	9.13 ± 0.39	<0.001
Ln(NT-proBNP, pg/ml)	4.66 ± 1.08	4.38 ± 0.90	0.001
hs-CRP [M(QR), mg/L]	2.15 (4.42)	0.88 (1.85)	<0.001
HCY (μmol/L)	14.97 ± 4.82	15.09 ± 4.46	0.866

### Echocardiography and HRV

We analyzed echocardiographic indicators between the declined HRV and normal HRV groups. The data showed that echocardiographic cardiac structure indicator LAD (35.40 ± 4.56 mm *vs.* 34.47 ± 4.56 mm, *p* = 0.015) was significantly increased, and cardiac function index E/A (0.78 ± 0.22 *vs.* 0.83 ± 0.26, *p* = 0.029) was significantly decreased in the declined HRV group (**Table 2**). However, LVEF, E/e′, and most cardiac structure indicators did not differ between groups.

**Table 2:** Echocardiographic examinations of subjects categorized by SDNN.

Echocardiographic examinations	HRV		*p*
	SDNN <100 ms	100 ms≤ SDNN ≤180 ms	
	(n = 250)	(n = 425)	
AOD (mm)	32.89 ± 3.57	32.56 ± 3.85	0.301
LAD (mm)	35.40 ± 4.56	34.47 ± 4.56	0.015
LVDd (mm)	44.69 ± 4.95	44.73 ± 3.63	0.354
IVSd (mm)	9.89 ± 1.45	9.74 ± 1.21	0.160
MPAD (mm)	20.90 ± 2.33	20.89 ± 2.26	0.960
RADd (mm)	33.56 ± 3.40	33.95 ± 3.70	0.202
RVDd (mm)	20.30 ± 2.09	20.43 ± 2.16	0.471
LVPWd (mm)	9.72 ± 1.14	9.64 ± 1.11	0.403
LVEF (%)	58.56 ± 3.99	59.25 ± 2.42	0.072
E/e’	11.10 ± 3.45	10.80 ± 2.69	0.528
E/A	0.78 ± 0.22	0.83 ± 0.26	0.029

Furthermore, bivariate linear correlation analysis showed that the E/A level was significantly and positively correlated with HRV parameters including SDNN (*r* = 0.148, *p* < 0.001), SDNN index (*r* = 0.164, *p* < 0.001), and Triangle index (*r* = 0.088, *p* = 0.030), but not with rMSSD and LF/HF (**Table 3**).

**Table 3:** Correlation coefficients between CaA, E/A, and HRV variables.

HRV	CaA		E/A	
	*r*	*p*	*r*	*p*
SDNN	0.413	<0.001	0.148	<0.001
SDNN Index	0.209	<0.001	0.164	<0.001
rMSSD	0.121	0.002	0.028	0.493
Triangle index	0.218	<0.001	0.088	0.030
LF/HF	-0.018	0.645	0.033	0.415

### Serum Calcium and HRV

To determine the variables associated with SDNN, logistic regression analysis was developed to include CaA, age, HbA1c, HDL-C, LDL-C, Ln(NT-proBNP), and hs-CRP. Declined HRV (SDNN <100 ms) was significantly associated with CaA [OR (95% CI), 0.056 (0.028–0.110), *p* < 0.001], age [OR (95% CI), 1.018 (1.001–1.035), *p* = 0.037], HbA1c [OR (95% CI), 1.253 (1.109–1.416), *p* < 0.001], Ln(NT-proBNP) [OR (95% CI), 1.230 (1.017–1.488), *p* = 0.033], and hs-CRP [OR (95% CI), 1.031 (1.013–1.049), *p* = 0.001] (**Table 4**). Bivariate linear correlation analysis showed that the CaA level was significantly and positively correlated with HRV parameters including SDNN (*r* = 0.413, *p* < 0.001), SDNN index (*r* = 0.209, *p* < 0.001), rMSSD (*r* = 0.121, *p* = 0.002), and Triangle index (*r* = 0.218, *p* < 0.001), but not with the LF/HF (**Table 3**).

**Table 4:** Logistic regression analysis of risk factors for SDNN.

	B	S.E.	Wald	df	p value	OR	95% CI
Age	0.018	0.009	4.350	1	0.037	1.018	1.001–1.035
HbA1c	0.226	0.062	13.076	1	<0.001	1.253	1.109–1.416
HDL-C	-0.396	0.366	1.174	1	0.279	0.673	0.328–1.378
LDL-C	-0.154	0.111	1.920	1	0.166	0.858	0.690–1.066
CaA	-2.884	0.346	69.532	1	<0.001	0.056	0.028–0.110
Ln(NT-proBNP)	0.207	0.097	4.538	1	0.033	1.230	1.017–1.488
hs-CRP	0.031	0.009	12.021	1	0.001	1.031	1.013–1.049

**Figure 1:**
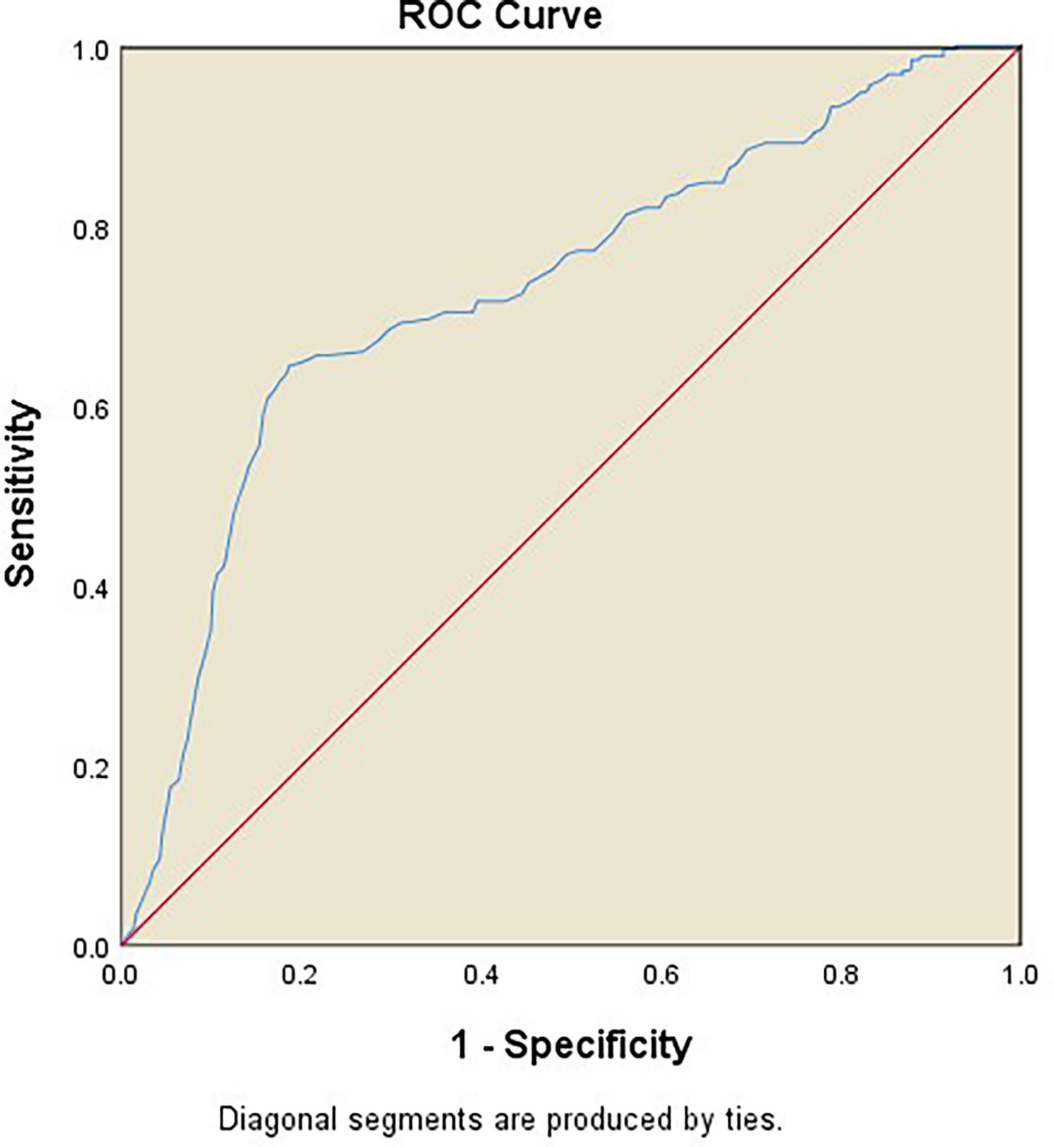
ROC curves of CaA on prediction of HRV. The AUC in the ROC curve was 0.730 [95% CI (0.750–0.815), *p* < 0.001]. The cutoff value of CaA was 8.87 mg/dl (sensitivity 0.644, specificity 0.814).

To evaluate the predictive performance of CaA on HRV, the AUC in the ROC curve was calculated, which was 0.730 [95% CI (0.750–0.815), *p* < 0.001]. The cutoff value of CaA was 8.87 mg/dl (sensitivity 0.644, specificity 0.814) (**Figure 1**). We divided these patients into two groups (CaA level <8.87 mg/dl and ≥8.87 mg/dl) based on the cutoff value. HRV parameters, including SDNN (*p* < 0.001), SDNN index (*p* < 0.001), rMSSD (*p* = 0.006), and Triangle index (*p* < 0.001), were significantly decreased in the group of lower calcium level (**Table 5**). However, LF/HF did not differ between the two groups.

**Table 5:** Relationship of serum calcium with HRV and cardiac function in T2DM patients.

	CaA (mg/dL)		*p*
	<8.87 (n = 242)	≥8.87 (n = 433)	
SDNN [M(QR), ms]	92 (34)	120 (37)	<0.001
SDNN index [M(QR)]	38 (17)	46 (22)	<0.001
rMSSD [M(QR), ms]	21 (14)	23 (15)	0.006
Triangle index [M(QR)]	23 (11)	26 (12)	<0.001
LF/HF	1.81 ± 1.37	1.92 ± 1.21	0.506
AOD (mm)	33.07 ± 3.65	32.46 ± 3.79	0.054
LAD (mm)	35.10 ± 4.51	34.66 ± 4.62	0.250
LVDd (mm)	45.22 ± 4.04	44.44 ± 4.21	0.026
IVSd (mm)	9.77 ± 1.22	9.81 ± 1.36	0.721
MPAD (mm)	20.89 ± 2.52	20.89 ± 2.15	0.663
RADd (mm)	34.19 ± 3.23	33.60 ± 3.77	0.050
RVDd (mm)	20.22 ± 1.97	20.47 ± 2.22	0.155
LVPWd (mm)	9.66 ± 1.10	9.68 ± 1.13	0.768
LVEF (%)	58.67 ± 3.67	59.17 ± 2.73	0.373
E/e′	10.94 ± 2.90	10.90 ± 3.07	0.940
E/A	0.79 ± 0.24	0.82 ± 0.26	0.251
Ln(NT-proBNP, pg/ml)	4.57 ± 0.95	4.44 ± 0.10	0.113
FPG (mmol/L)	7.77 ± 3.38	7.71 ± 3.17	0.827
HbA1c (%)	7.86 ± 1.36	7.66 ± 1.54	0.093
TyG index	9.06 ± 0.71	9.13 ± 0.74	0.195

The multivariate logistic regression analysis shows the ORs (95% CI) for declined HRV according to the two groups of CaA levels. In contrast to subjects with CaA level ≥8.87 mg/dl, there was a significantly increased risk of declined HRV in subjects with CaA level <8.87 mg/dl [OR (95% CI), 0.049 (0.024–0.099), *p* < 0.001] after adjusting for possible confounding factors including creatinine, serum phosphate, age, gender, smoking, drinking, HbA1c, Ln(NT-proBNP), hs-CRP, TyG index, dyslipidemia, hypertension, coronary heart disease, carotid atherosclerosis, and the use of hypertension medication.

### Serum Calcium and Cardiac Function

We analyzed echocardiographic indicators and cardiac function between the two groups (CaA level <8.87 and ≥8.87 mg/dl). LVDd (45.22 ± 4.04 mm *vs*. 44.44 ± 4.21 mm, *p* = 0.026) was significantly increased in the group of lower calcium level. However, cardiac function indexes (LVEF, E/e′, E/A, and NT-proBNP) and most cardiac structure indicators (AOD, LAD, IVSDd, MPAD, RADd, RVDd, and LVPWd) did not differ between the two groups (**Table 5**). There were no significant differences in FBG, HbA1c, and TyG index between the two groups.

## Discussion

Previous studies confirmed that the independent predictors of CAN in T2DM were age, HbA1c, BMI, and triglycerides (8, 25, 26). However, these features as determinants of the autonomic dysfunction are insufficient. In this study, we showed that T2DM patients with CAN (declined HRV) had a significantly lower CaA level, and poorer cardiac function, than those with normal HRV. On the other hand, T2DM patients with a lower CaA level had significantly decreased HRV parameters, but cardiac function did not differ between the two groups. Therefore, for the first time we showed that CaA level, even within the normal range, was independently associated with declined HRV in T2DM after adjustment for other confounding factors, which suggests that decreased serum calcium level may be an effective predictor of CAN in T2DM.

Early studies found that decreased HRV was associated with an increased risk of cardiovascular death (27). HRV is now considered to be a reliable method to evaluate CAN in diabetes (8, 25, 26). A large proportion of diabetic patients have autonomic dysfunction, which is an independent predictor of vascular dysfunction. The prevalence of CAN in diabetic patients varies from 7.7% in newly diagnosed diabetic patients to 90% in patients planning pancreas transplant (28). In our study, 37.0% of the T2DM patients were accompanied by declined HRV, which is less than the incidence rate of CAN in the DCCT/EDIC (25). The reason of this may be the different exclusion criteria and different diabetes population between these two studies.

Among HRV parameters, SDNN reflects the whole 24-h HRV and can be used to be a marker of overall autonomic modulation (29). Our study showed that age was negatively correlated with SDNN, which matches previous studies that age was an important prognostic factor for HRV (30). Hs-CRP was also a risk factor for declined HRV in T2DM, indicating that inflammation may be involved in the initiation and progression of CAN in T2DM (31, 32). Our data also indicated that HbA1c was the important risk factor of declined HRV, indicating that glycemic control may be an important means of therapy for reducing CAN risk in diabetes. From the ACCORD Study (33), it showed that CAN patients with decreased SDNN values had an increased risk of mortality during follow-up. However, the increased mortality of T2DM was independent of glycemia control. T2DM is associated with abnormal cardiovascular autonomic function (34). Our study showed that T2DM patients with declined HRV had a significantly poorer cardiac function than those with normal HRV, suggesting that declined HRV in T2DM increases the risk of heart failure. We further analyze the linear relationship between E/A and HRV indicators. It showed that E/A was significantly and positively correlated with time-domain HRV indices including SDNN, SDNN index, and triangle index, which suggested that declined HRV in T2DM was associated with cardiac diastolic dysfunction.

In our study, only T2DM patients with normocalcemia were included. We showed that the cutoff value of CaA to predict risk of declined HRV was 8.87 mg/dl. T2DM patients with CaA level <8.87 mg/dl had a significant declined HRV compared to patients with CaA level ≥8.87 mg/dl. It indicates that the fluctuation of serum calcium in the normal range still affects HRV. However, cardiac function was not significantly different between the two groups. A similar study showed that albumin-adjusted serum calcium was positively associated with an increased risk of left ventricular hypertrophy in T2DM patients (35). The different results may be due to different inclusion and exclusion criteria.

Our study provided not only a convenient alternative method to assess early CAN patients in diabetes but also a basis for selection of suitable anti-glycemic agents. For example, sodium-glucose cotransporter 2 (SGLT2) inhibitors are a class of glucose-lowering agents for the treatment of type 2 diabetes by selectively inhibiting renal glucose reabsorption and increasing urinary glucose excretion. Clinical studies have found that SGLT2 inhibitors improve heart failure and cardiovascular outcomes in patients with T2DM (36). Evidence also showed that SGLT2 inhibitors may slightly increase calcium levels by reducing urinary calcium excretion (37, 38). This evidence suggests that SGLT2 inhibitors may be beneficiary for T2DM patients with CAN, but further studies are needed.

Although there have been no studies that focused on the direct mechanism responsible for the effects of calcium on HRV and cardiac function in diabetic patients, we infer possible mechanisms including reduced vascular reactiveness, increased intima-media thickness, and endothelium dysfunction (39). Besides, calcium could be involved in the sympathetic nervous activation *via* insulin resistance and its associated compensatory hyperinsulinemia, contributing to diminished HRV in diabetic patients, resulting in cardiac function impairment such as left ventricular hypertrophy or diastolic dysfunction (40). However, our study did not show a correlation between serum calcium levels and glycemic control (data not shown).

Several limitations of this study should be noted. First, the enrolled patients with diabetes are relatively mild owing to strict exclusion criteria. The conclusions of this study may be inconsistent with other studies which included more severe patients. Second, most of the patients are unavailable for serum parathyroid hormone and vitamin D levels. Although no hypercalcemia or hypocalcemia individuals were excluded, it is impossible to completely exclude potential confounding factors. Third, these results were based on serum calcium at admission, which may not be representative for the patients.

## Conclusions

Declined HRV is associated with a lower CaA level and worse cardiac function in patients with T2DM. A lower serum calcium level in the normal range was independently associated with decreased HRV in T2DM patients. Routinely monitoring calcium level may help to identify/screen high-risk CAN patients in T2DM and facilitate its early intervention.

## Data Availability Statement

The raw data supporting the conclusions of this article will be made available by the authors, without undue reservation.

## Ethics Statement

The studies involving human participants were reviewed and approved by the ethical review board of Renmin Hospital of Wuhan University. Written informed consent for participation was not required for this study in accordance with the national legislation and the institutional requirements.

## Author Contributions

JW concepted and designed the research. JW, KL, YY, DL, LW, FZ, AY, SW, and JL contributed to the acquisition of data. JW, ZX, and LG contributed to the analysis, interpretation of data, and drafting of the article. ZX and LG revised the manuscript critically for important intellectual content. All authors contributed to the article and approved the submitted version.

## Funding

This study was supported by the National Natural Science Foundation of China (Project #81571376 to LG), Diabetes Study Fund from the Chinese Medical Association (Project # 13060906481 to LG), the Fundamental Research Funds for the Central Universities (Project # 2042020kf1079 to LG), and Hubei Province Natural Science Foundation (Project # 2021CFB448 to ZX).

## Conflict of Interest

The authors declare that the research was conducted in the absence of any commercial or financial relationships that could be construed as a potential conflict of interest.

## Publisher’s Note

All claims expressed in this article are solely those of the authors and do not necessarily represent those of their affiliated organizations, or those of the publisher, the editors and the reviewers. Any product that may be evaluated in this article, or claim that may be made by its manufacturer, is not guaranteed or endorsed by the publisher.
